# A retrospective cohort study of body mass index and survival in HIV infected patients with and without TB co-infection

**DOI:** 10.1186/s40249-018-0418-3

**Published:** 2018-04-25

**Authors:** Kogieleum Naidoo, Nonhlanhla Yende-Zuma, Stanton Augustine

**Affiliations:** 1 Centre for the AIDS Programme of Research in South Africa (CAPRISA), Nelson R Mandela School of Medicine, University of KwaZulu-Natal, 2nd Floor Doris Duke Medical Research Institute, Private Bag X7, Durban, Congella 4013 South Africa; 2 MRC-CAPRISA HIV-TB Pathogenesis and Treatment Research Unit, Doris Duke Medical Research Institute, University of KwaZulu-Natal, Durban, South Africa

**Keywords:** HIV, Tuberculosis, BMI, ART, Africa, KwaZulu-Natal, KZN, South Africa

## Abstract

**Background:**

High early morbidity and mortality following antiretroviral therapy (ART) initiation has been a distinguishing feature of ART programmes in resource limited settings (RLS) compared to high-income countries. This study assessed how well body mass index (BMI: kg/m^2^) correlated with survival among HIV infected patients with and without TB co-infection.

**Methods:**

We retrospectively evaluated clinical data from 1000 HIV infected patients, among whom 389 were also co-infected with TB, between January 2008 and December 2010, in KwaZulu-Natal, South Africa.

**Results:**

Among 948 patients eligible for analysis, 15.7% (149/948) were underweight (< 18.50), 55.9% (530/948) had normal BMI (≥18.50–24.90), 18.7% (177/948) were overweight (25.00–29.00) and 9.7% (92/948) were obese (≥30.00). Irrespective of TB status, underweight patients, had significantly higher risk of death compared to those with normal BMI at baseline (aHR = 2.9; 95% *CI*: 1.5–5.7; *P* = 0.002).

**Conclusions:**

Irrespective of TB co-infection, low BMI correlated with mortality in HIV infected patients.

**Trial registration:**

UKZN Biomedical Research Ethics Committee Reference number E 248/05, 23 September 2005.

**Electronic supplementary material:**

The online version of this article (10.1186/s40249-018-0418-3) contains supplementary material, which is available to authorized users.

## Multilingual abstracts

Please see Additional file 1 for translations of the abstract into the five official working languages of the United Nations.

## Background

Human immunodeficiency virus (HIV) infection creates serious health and developmental challenges, especially in resource limited settings [1]. Since first reported, approximately 36 million people have died from disease associated with acquired immune deficiency syndrome (AIDS), and another 35.3 million are currently living with HIV/AIDS, worldwide [1]. While the introduction and scale-up of ART has reduced overall mortality, high early morbidity and mortality following ART initiation, remains a distinguishing feature of ART programs in sub-Saharan Africa [2–4]. Furthermore, the universal test and treat strategy enables ART access irrespective of CD4^+^ count [5], which may reduce numbers of patients that present for care with advanced HIV disease, including a low BMI.

Despite this strategy, we still find higher rates of morbidity and mortality, driven largely by late presentation for ART [6]. This is exacerbated by structural resource limitations related to inadequate clinical and diagnostic infrastructure, fewer qualified human resources, and increased workload due to large patient volumes [4]. Currently laboratory based CD4^+^ count and HIV viral load tests are the only tools used in monitoring therapeutic outcomes in patients. The high cost and limited availability of personnel and infrastructure supporting use of these tools, compounded by the need for frequent patient visits to access laboratory results, further complicate provision of adequate monitoring of treatment outcomes in disease endemic settings [7]. While the world waits for more affordable point of care diagnostics, the need for low cost, routinely available non-invasive clinical measurements for efficient triage of patients at high risk of adverse clinical outcomes, remains a high priority [7, 8].

Weight loss and malnutrition are frequently described as clinical markers for poor prognosis among HIV infected patients, especially in the pre-ART era [8–13]. Severe weight loss is most often due to HIV infection itself, or an associated co-morbidity [7, 9–13]. Published data from low-income countries demonstrate that mortality was more strongly associated with weight loss combined with co-morbid infections compared to just CD4^+^ count threshold [2]. While data from South Africa showed that patients with both prevalent and incident tuberculosis (TB) had a two-fold greater risk of mortality despite ART, the role of baseline BMI on clinical outcomes had not been evaluated [2, 14].

In settings with limited diagnostic capacity, body mass index (BMI- kg/m^2^), is likely to represent a number of difficult-to-diagnose conditions such as intestinal malabsorption, chronic gut opportunistic infections, increased metabolism including others [8, 9]. BMI, which provides an estimate of healthy body weight in relation to height, may be a useful surrogate marker of prognosis in HIV-TB co-infection, with potential to simplify the identification of patients at high risk of death through ease of use [7]. A review recognized BMI as a useful predictor of AIDS disease progression in the pre-ART era, citing its potential use for determining survival outcomes in patients initiating ART with BMI < 18.00 [11].

Improved use of simple clinical prognostic factors would enable early identification and triage of patients for closer clinical observation. This would facilitate early intervention for patients at high risk for adverse clinical outcomes and mortality [7, 8, 10–13]. This study aimed to assess whether there is an association between BMI and survival among HIV infected patients initiating ART, including a group also co-infected with TB, in KwaZulu-Natal, South Africa.

## Methods

Centre for the AIDS Programme of Research in South Africa (CAPRISA) started a PEPFAR-funded CAPRISA AIDS Treatment Programme (CAT) where ambulant HIV infected, ART naïve adult patients were initiated on ART between June 2004 and August 2013. While 1807 patients were initiated on ART between 2008 and 2010, due to a limited budget, we randomly selected 1000 files from this cohort for review. The aim of conducting this retrospective cohort study was to evaluate whether BMI predicted survival in HIV infected patients with and without baseline prevalent TB. Enrolment was voluntary, and all patients were treated as per current South African Government HIV/AIDS and Tuberculosis Treatment Guidelines [15, 16]. Routine demographic, laboratory and clinical data were recorded at baseline and at monthly follow-up visits. Standard TB outcome definitions were used as per national guidelines, and HIV viral suppression was defined as a viral load of < 400 copies/ml [15, 16]. We used a random number generator to randomly select those for inclusion into this analysis after numbering patients based on chronology of enrolment [17]. Follow-up time was calculated from ART initiation to date of death, withdrawal from the study, loss to follow-up, transfer out or censored at 18 months post-ART initiation, whichever occurred first. Body mass index was calculated as per World Health Organization (WHO) definition i.e. weight (kg)/height (m) ^2^ [18]. Patients were grouped on their baseline BMI into four categories: < 18.50 (underweight), 18.50–24.99 (normal), 25.00–29.99 (overweight), and ≥ 30.00 (obese). Primary endpoints included mortality. Secondary endpoints included TB and HIV outcomes. This study was approved by the Biomedical Research Ethics Committee (BREC, reference number: E248/05) of the University of KwaZulu-Natal.

### Statistical analysis

Data were presented as means with standard deviation, medians with interquartile range for continuous variables; and proportions for categorical data. Where appropriate, Fisher–Freeman–Halton test was used to analyse categorical data, and one-way analysis of variance test or Kruskal–Wallis test used for analysis of continuous data. Poisson approximations were used to calculate confidence intervals (*CI*s) for mortality rates and mortality rate ratios. Log-rank test was used to compare survival distributions of BMI categories. Multivariate proportional hazards regression was used to estimate the association between mortality and BMI, adjusting for age, gender, CD4^+^ count, TB status and WHO stage. Statistical significance was defined as a two-sided *P*-value less than 0.05. Statistical analysis was performed using SAS (version 9.4; SAS Institute Inc., Cary, NC).

## Results

### Baseline

A total of 1000 patients were included in the analyses (500 per treatment site) from a pool of 1807. Since height or weight measurements were not available in 52 patients, only 948 were included in the final analysis.

We found 15.7% (149/948) patients were underweight, 55.9% (530/948) had normal BMI, 18.7% (177/948) were overweight and 9.7% (92/948) were obese. Female patients predominated in the overweight (83.1%, 147/177) and obese (90.1%, 82/92) categories compared to male patients (*P* < 0.001). Median (IQR) baseline CD4^+^ counts were significantly lower in underweight patients (96 cells/mm^3^) compared to normal (132 cells/mm^3^), overweight (150 cells/mm^3^) and obese (140 cells/mm^3^) patients (*P* = 0.002), respectively. In addition, obese patients had significantly lower baseline viral loads (4.5 log copies/ml) than underweight (5.2 log copies/ml), normal (5.0 log copies/ml) and overweight (4.7 log copies/ml) patients, respectively (*P* < 0.001). TB prevalence at baseline among patients in each of the BMI categories was 55.7% (83/149), 43.2% (229/530), 32.8% (58/177) and 20.7% (19/92), respectively (*P* < 0.001) (Table 1).

**Table 1:** Baseline and follow-up characteristics of patients enrolled onto ART

Variable	BMI < 18.5 (*N* = 149)	BMI (18.5–24.99) (*N* = 530)	BMI (25–29.99) (*N* = 177)	BMI (≥30) (*N* = 92)	*P*-value
Baseline					
Median (IQR), age (years)^a^	33 (29–39)	33.5 (29–40)	35 (28–42)	36 (30–43)	0.278
Female, %(*n*)^b^	36.9 (55)	49.6 (263)	83.1 (147)	90.1 (82)	< 0.001
WHO stage 1–3,% (*n*)^c^	69.8 (104)	84.9 (450)	84.8 (150)	93.4 (85)	< 0.001
WHO stage 4, % (*n*)^c^	30.2 (45)	15.1 (80)	15.3 (27)	6.6 (6)	
CD4^+^, Median (IQR)^d^ (cells /mm^3^)	96 (35–187)	132 (66–196)	150 (75–224)	140 (81–214)	0.002
Mean (*SD*) viral load (log copies/ml)^e^	5.2 (0.9)	5.0 (0.8)	4.7 (0.9)	4.5 (1.0)	< 0.001
Tuberculosis at baseline	55.7 (83)	43.2 (229)	32.8 (58)	20.7 (19)	< 0.001
Follow-up					
CD4^+^ increase > 50: baseline to 6 months, %(*n*) (*N* = 741)	78.3 (83)	80.4 (328)	74.0 (111)	70.1 (54)	0.141
CD4^+^ increase > 50: baseline to 12 months, %(*n*) (*N* = 512)	85.0 (68)	85.7 (252)	83.2 (79)	83.7 (36)	0.935
CD4^+^ increase > 50: baseline to 18 months, %(*n*) (*N* = 237)	85.7 (24)	91.4 (127)	91.7 (44)	86.4 (19)	0.631
Undetectable viral load at 6 months, % (*n*) (*N* = 810)	90.7 (107)	93.7 (419)	93.8 (152)	92.7 (77)	0.679
Undetectable viral load at 12 months, % (*n*) (*N* = 524)	91.5 (75)	95.0 (283)	96.0 (96)	90.9 (40)	0.401
Undetectable viral load at 18 months, % (*n*) (*N* = 242)	82.1 (23)	93.0 (132)	93.9 (46)	87.0 (20)	0.195

*SD*: Standard deviation IQR: Interquartile range

^a^2 Missing age ^b^1 Missing WHO stage ^c^Missing gender ^d^112 Missing CD4^+^ count ^e^92 Missing viral load

### HIV and TB outcomes by baseline BMI

Despite statistically significant differences in mean log viral load at baseline across the BMI categories, viral load suppression rates on ART were not significantly different at 6, 12 and 18 months follow up (Table 1). TB treatment outcomes of cure and successful TB treatment completion was 74.3%, 82.9%, 90% and 77.8% in the underweight, normal, overweight and obese categories, respectively (*P* = 0.489).

### Mortality

**Fig. 1:**
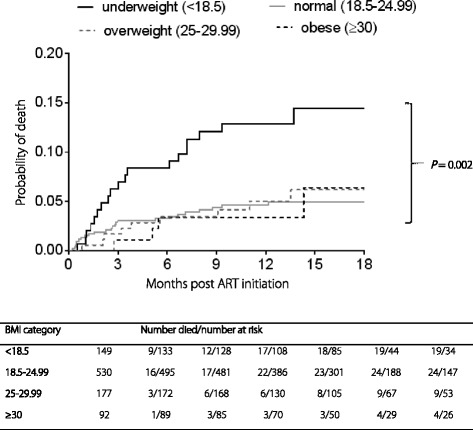
Kaplan-Meier estimates of cumulative probability of death, by BMI group

During a median follow-up of 12.8 months (interquartile range [IQR]) 8.8 to 18 months, a total of 56 deaths occurred over 974.2 person-years of follow-up. Underweight patients had higher overall mortality rates of 13.0 (95% *CI*: 7.8–20.3) per 100 person-years, compared to patients that were normal 4.4 (95% *CI*: 2.8–6.6); overweight 4.7 (95% *CI*: 2.2–9.1); and obese 4.2 (95% *CI*: 1.1–10.8) per 100 person-years, respectively (*P* = 0.002) (Table 2, Fig. 1). Mortality was significantly different among the BMI categories for TB patients (Table 2). We did not observe significant differences in mortality rates among those with and without TB within each of the BMI strata (Table 2).

**Table 2:** Mortality rates for different BMI classification

	BMI < 18.5		BMI 18.5–24.99		BMI 25–29.99		BMI ≥ 30		*P*-value**
	Deaths/person-years	Mortality rate (95% *CI*)	Deaths/person-years	Mortality rate (95% *CI*)	Deaths/person-years	Mortality rate (95% *CI*)	Deaths/person-years	Mortality rate (95% *CI*)	
All	19/146.2	13.0 (7.8–20.3)	24/545.0	4.4 (2.8–6.6)	9/188.2	4.7 (2.2–9.1)	4/94.8	4.2 (1.1–10.8)	0.002
TB	11/80.8	13.6 (6.8–24.5)	7/240.6	2.9 (1.2–6.0)	5/60.7	8.2 (2.7–19.2)	1/19.2	5.2 (0.1–29.1)	0.007
No TB	8/65.4	12.2 (5.3–24.1)	17/304.4	5.6 (3.2–8.9)	4/127.5	3.1 (0.9–8.0)	3/75.7	4.0 (0.8–11.6)	0.076
*P*-value*		0.818		0.147		0.150		0.851	

*BMI* Body Mass Index

*P-value comparing TB and no TB group within each BMI stratum

**P-value comparing BMI strata across each TB category

The multivariate proportional hazards regression model showed that underweight patients had significantly higher risk of death compared to those with normal BMI at baseline (aHR = 2.9; 95% *CI*: 1.5–5.7; *P* = 0.002) (Fig. 1). These results were adjusted for baseline characteristics such as age, gender, CD4^+^ count, TB status and WHO stage of HIV. Among the variables adjusted for, only WHO stage was a statistically significant predictor for mortality, (aHR = 2.04; 95% *CI*: 1.07–3.89; *P* = 0.029) (Table 3).

**Table 3:** Analysis of baseline factors associated with mortality

Variable at ART initiation		Univariate		Multivariate	
		HR (95% *CI*)	*P*-value	aHR (95% *CI*)	*P*-value
BMI group, kg/m^2^ (ref: 18.5–24.99)	< 18.5	2.91 (1.6–5.32)	0.001	2.93 (1.51–5.7)	0.002
	25–29.99	1.09 (0.51–2.35)	0.825	1.25 (0.51–3.09)	0.624
	≥30	0.95 (0.33–2.73)	0.918	1.14 (0.32–4.04)	0.841
Age (per 5–year increase)		1.09 (0.95–1.25)	0.238	1.1 (0.94–1.29)	0.232
Gender (ref: female)	Male	1.85 (1.09–3.14)	0.023	1.42 (0.74–2.72)	0.290
CD4^+^ cell count (per 50 cells/mm^3^ increase)		0.87 (0.74–1.02)	0.091	0.92 (0.8–1.06)	0.262
WHO stage (ref: 1–3)	Stage 4	2.53 (1.45–4.44)	0.001	2.04 (1.07–3.89)	0.029
Tuberculosis (ref: No)	Yes	1.05 (0.62–1.78)	0.857	0.99 (0.54–1.81)	0.976

*aHR* Adjusted hazard ratio

## Discussion

In high burden TB-HIV co-endemic settings, that also have limited diagnostic capacity, an inexpensive, simple and widely available triage tool is essential to identify patients at high risk for morbidity and early mortality. While most studies have examined the role of BMI on survival in HIV mono-infected patients, we assessed the association of BMI and survival in HIV infected patients, some of whom were co-infected with TB. Our data shows that baseline BMI is a strong and independent predictor of mortality in HIV infected patients, irrespective of TB status. We highlight that patients who presented with baseline BMI < 18.50 experienced 3-fold higher mortality rates than those in higher BMI categories, regardless of tuberculosis status. It is also important to note that mortality rates were similar in all three BMI categories above 18.50, irrespective of tuberculosis co-infection.

HIV infection profoundly affects nutritional status because it is associated with poor appetite, impaired nutrient absorption, increased basal metabolic rate and opportunistic infections [2, 8]. Studies in industrialized countries have shown a correlation between poor nutritional status prior to ART initiation and clinical progression [9], whilst studies from Africa have demonstrated a correlation between low baseline BMI and subsequent higher mortality. Furthermore, data from a Malawian cohort of tuberculosis patients, among whom 80% were HIV co-infected, found that a baseline BMI < 17.00 independently predicted mortality within the first 4 weeks of starting tuberculosis treatment. Baseline BMI in this cohort, is similar to published data from African patients, but lower than BMI in studies from European countries [7, 9].

In the study by Forouzanfar et al. the proportion of females was 4-fold higher compared to males in the > 30.00 BMI group [19]. In 2008, the average BMI in South Africa was 26.9 kg/m^2^ among males (vs. a world average of 23.80 kg/m^2^), and 29.50 kg/m^2^ among females (vs. a world average of 24.1 kg/m^2^) [20]. Interestingly, a recent meta-analysis from 1990 to 2015 found that unsafe sex and high BMI was ranked first and second leading risk factors for early death and disability in South Africa. In a maturing HIV epidemic, the likely impact of high BMI on non-communicable diseases related morbidity and mortality is very concerning given the large numbers of patients receiving chronic ART.

We could not demonstrate an association between CD4^+^ counts and BMI, as the range of CD4^+^ counts in our study was small due to the ART eligibility criteria. We do however show that CD4^+^ count was not an independent predictor of mortality.

In a study conducted in four sub-Saharan countries investigating timing of ART in TB-HIV co-infected patients with CD4^+^ > 220 cells/mm^3^, overall mortality rates were low, despite a baseline BMI of < 18.50 in approximately 40% of patients [12]. A study evaluating correlation between BMI and CD4^+^ count gains found that baseline BMI predicted change in CD4^+^ count after week 96. Relative to men with normal BMI, overweight and obese men had higher CD4^+^ count increases. It is clear from these studies that low baseline BMI together with low CD4 counts predict mortality, while high BMI is associated with larger CD4^+^ counts increases, the latter only due to the impact of ART [12].

Criteria used to clinically stage HIV disease, and diagnose AIDS defining illnesses, are internationally accepted but are often difficult to apply in resource-poor environments, as they require sophisticated and expensive investigations to make definitive diagnoses [21]. Furthermore, AIDS care is usually delivered by nurses, who are exposed to limited clinical and diagnostic training reducing their ability to appropriately identify and up-refer patients at high risk for poor outcomes. Thus, there is clearly a need for less sophisticated prognostic clinical indicators suitable for task-shifted activities conducted by frontline health care workers in resource limited settings.

We acknowledge several limitations of the study. Data used in this analysis was routinely collected programmatic data, which was flawed by missing data variables on height and weight, reducing our analysable sample. Autopsies are not routinely performed for HIV infected patients that demise. Autopsy data would have improved our understanding of the causes of mortality by the different BMI categories. While we see similar mortality rates among obese patients compared to those with a BMI between 18.50–29.90, these findings need further evaluation given the very small sample size in the obese category. Furthermore, we followed-up patients for 18 months only. Longer follow-up may have provided valuable information on BMI changes following chronic ART, and a further understanding of BMI category on risk of non-communicable disease in clade C HIV infected patients.

## Conclusions

BMI is associated with mortality in HIV infected patients initiating ART and is regarded as an affordable, low-technology, prognostic indicator; independent of age, sex, CD4^+^ count, or HIV type. Further prospective studies are indicated to evaluate whether BMI could be used to inform intervention decisions, including decisions of when to refer patients for diagnostic work-up or admission.

## Additional file

Additional file 1: Multilingual abstracts in the five official working languages of the United Nations. (PDF 443 kb)

## Abbreviations

AIDS: Acquired immune deficiency syndrome
ART: Antiretroviral therapy
BMI: Body mass index (BMI kg/m^2^)
HIV: Human immunodeficiency virus
TB: Tuberculosis
