# Supplementary material for: A retrospective cohort study of body mass index and survival in HIV infected patients with and without TB co-infection
Source: Infect Dis Poverty. 2018 Apr 25;7:35. doi: 10.1186/s40249-018-0418-3 (PMC5937835; doi:10.1186/s40249-018-0418-3)
Supplement: Supplementary file 1 — Multilingual abstracts in the five official working languages of the United Nations. (PDF 443 kb) [file 40249_2018_418_MOESM1_ESM.pdf]

Translation of the abstract into the five official working languages of the United Nations

أُجريت دراسة أترابية استعادية حول مؤشر كتلة الجسم ومعدلات البقاء على قيد الحياة بين المرضى المصابين بفيروس نقص المناعة البشرية الذين يعانون أيضاً من داء السل والمصابين بالفيروس فقط.

كوجيليوم نايدو، ونونلانهلا يندي - زوما، وستانتون أوغسطين

الملخص

المعلومات الأساسية: كانت معدلات الاعتلال المبكرة والوفيات المرتفعة بعد بدء العلاج المضاد للفيروسات الرجعية سمة مميزة لبرامج المعالجة بمضادات الفيروسات الرجعية في البيئات محدودة الموارد (RLS) مقارنة بالبلدان ذات الدخل المرتفع. تُقيِّم هذه الدراسة العلاقة الوثيقة بين مؤشر كتلة الجسم (كجم/م ^{2}) ومعدلات البقاء على قيد الحياة بين المرضى المصابين بفيروس نقص المناعة البشرية، وبعض المصابين بفيروس نقص المناعة البشرية وداء السل في آن واحد.

الأساليب المستخدمة: قمنا بتقييم البيانات السريرية بأثر رجعي لما يقدر بـ 1000 مريض مصاب بفيروس نقص المناعة البشرية، من بينهم 389 مريض كانوا مصابين بالسل كذلك، في الفترة بين يناير 2008 وديسمبر عام 2010، في كوازولو ناتال، جنوب أفريقيا.

النتائج: من بين 948 مريضاً مؤهلاً للتحليل كان 15.7٪ (948/149) من المرضى يعانون من نقص الوزن (< 18.5)،وحوالي 55.9٪ (948/530) من المرضى لديهم معدلات عادية لمؤشر كتلة الجسم (≤ 18.5-24.9)، وكان 18.7٪ (948/177) من المرضى يعانون من زيادة الوزن (25- 29) و 9.7٪ (948/92) من المرضى يعانون من السمنة المفرطة (≤ 30). وبغض النظر عن الإصابة بمرض السل، فإن المرضى الذين يعانون من نقص الوزن كان لديهم معدلات خطر بالموت أعلى بكثير مقارنة بالمرضى الذين لديهم مؤشر كتلة الجسم العادي عند المعدلات الأساسية (aHR: 2.9; 95% CI: 1.5 – 5.7; P = 0.002).

النتائج: بغض النظر عن الإصابة بمرض السل، فإن انخفاض مؤشر كتلة الجسم يرتبط بمعدل الوفيات بين المرضى المصابين بفيروس نقص المناعة البشرية.

Translated from English version into Arabic by Maha Husaini and Mona M. Abd El-Monem, through

TRANSLATORS
WITHOUT BORDERS

体重指数与艾滋病毒和结核合并/非合并感染患者存活率的相关性回顾队列研究

Kogieleum Naidoo, Nonhlanhla Yende-Zuma, Stanton Augustine

摘要

引言：与高收入国家相比，HIV 感染者接受抗逆转录病毒治疗（ART）后的高早期发病率和死亡率一直是资源匮乏地区（RLS）ART 项目的显著特征。本研究评估了体重指数（BMI：kg/m^2）与 HIV 感染者（部分为 HIV 和结核合并感染者）存活率的相关性。

方法：我们回顾性地评估了 2008 年 1 月至 2010 年 12 月在南非纳塔尔省夸祖鲁的 1000 例 HIV 感染者的临床资料，其中 389 例为 HIV 和结核（TB）合并感染。

结果：在符合纳入标准的 948 例患者中，15.7%（149/948）体重偏低（BMI<18.5），55.9%（530/948）体重正常（BMI ≥ 18.5–24.9），18.7%（177/948）超重（BMI: 25–29），9.7%

（92/948）为肥胖（BMI≥30）。不计结核病状况时，体重偏低的 HIV 感染者的死亡风险明显高于体重正常的感染者（aHR = 2.9; 95％ *CI*：1.5–5.7; *P* = 0.002）。
结论：不考虑与 TB 合并感染，BMI 较低的 HIV 感染者死亡较高。

Translated from English version into Chinese by Jin Chen, edited by Pin Yang

Étude de cohorte rétrospective de l’indice de masse corporelle et de la survie des patients infectés par le VIH avec et sans co-infection tuberculeuse

Kogieleum Naidoo, Nonhlanhla Yende-Zuma, Stanton Augustine

Résumé

Contexte : Les régions à ressources limitées se distinguent des pays à revenus élevés par une morbidité et une mortalité précoces importantes peu après le début du traitement dans le cadre des programmes de traitement antirétroviral (TARV). Notre étude a évalué la corrélation entre l’indice de masse corporelle (IMC, en kg/m^2) et la survie parmi les patients infectés par le VIH et, pour certains, co-infectés par la tuberculose.

Méthodes : Nous avons évalué rétrospectivement les données cliniques de 1000 patients infectés par le VIH, dont 389 co-infectés par la tuberculose, relevées entre janvier 2008 et décembre 2010 dans la province du KwaZulu-Natal, en Afrique du Sud.

Résultats : Parmi les 948 patients retenus pour l’analyse, 15,7 % (149 sur 948) étaient en insuffisance pondérale (< 18,5), 55,9 % (530 sur 948) avaient un IMC normal (≥ 18,5 à 24,9), 18,7 % (177 sur 948) étaient en surpoids (25 à 29) et 9,7 % (92 sur 948) étaient obèses (≥ 30). Quel que soit le statut tuberculeux, les patients en insuffisance pondérale avaient un risque de décès significativement supérieur à ceux qui avaient un IMC normal au début de l’étude (RRa : 2,9 ; IC à 95 % : de 1,5 à 5,7 ; *P* = 0,002).

Conclusions : Quel que soit le statut de co-infection tuberculeuse, un IMC bas était corrélé à la mortalité chez les patients infectés par le VIH.

Translated from English version into French by Suzzane Assenat, through

Ретроспективное когортное исследование индекса массы тела и выживаемости ВИЧ-инфицированных пациентов как с присутствием коинфекции ТБ, так и в случае её отсутствия

Когиелеум Найду, Нонхланхла Йенде-Зума, Стантон Аугустин

Аннотация

Справочная информация: Высокий уровень ранней заболеваемости и смертности после инициации антиретровирусной терапии (АРТ) является отличительной чертой программ по АРТ в условиях ограниченных ресурсов в сравнении со странами с высоким уровнем дохода. В ходе настоящего исследования была произведена оценка взаимосвязи индекса массы тела (ИМТ: кг/м^2) с выживаемостью среди ВИЧ-инфицированных пациентов, некоторые из которых коинфицированы туберкулёзом.

Методы: За период с января 2008 года по декабрь 2010 года в Квазулу-Натале, ЮАР, была проведена ретроспективная оценка клинических данных по 1000 ВИЧ-инфицированных пациентов, среди которых 389 человек были коинфицированы ТБ.

Результаты: Среди 948 пациентов, удовлетворяющих условиям исследования, 15,7% (149/948) имели недостаточный вес (<18,5); у 55,9% (530/948) наблюдался нормальный ИМТ (≥18,5-24,9); 18,7% (177/948) страдали от избыточного веса (25-29) и 9,7% (92/948) страдали от ожирения (≥ 30). Вне зависимости от заражённости ТБ, у пациентов с недостаточным весом наблюдался значительно более высокий риск смерти в сравнении с теми, у кого показатель ИМТ был в норме на базовом уровне (aHR: 2,9; 95% *CI*: 1,5 – 5,7; *P* = 0,002).

Выводы: Вне зависимости от коинфицированности ТБ, низкий показатель ИМТ связан с уровнем смертности ВИЧ-инфицированных пациентов.

Translated from English version into Russian by Liudmila Tomanek and Oksana Rozhko, through

TRANSLATORS
WITHOUT BORDERS

Estudio retrospectivo de cohortes del índice de masa corporal y supervivencia en pacientes infectados por el VIH con y sin coinfección por TB

Kogieleum Naidoo, Nonhlanhla Yende-Zuma, Stanton Augustine

Resumen

Antecedentes: La alta morbilidad y mortalidad temprana luego de iniciada la terapia antirretroviral (ART por sus siglas en inglés) ha sido una característica distintiva de los programas de ART en entornos de recursos limitados, en comparación con los países de elevados ingresos. Este estudio evaluó qué tan bien se correlaciona el índice de masa corporal (IMC: kg/m^2) con la supervivencia en pacientes infectados con el VIH, algunos también infectados con tuberculosis.

Métodos: Evaluamos de forma retrospectiva los datos clínicos de 1000 pacientes infectados con el VIH, entre los cuales 389 también estaban infectados con TB, entre enero de 2008 y diciembre de 2010, en KwaZulu-Natal, Sudáfrica.

Resultados: Entre los 948 pacientes elegibles para el análisis, 15,7% (149/948) tenían bajo peso (<18,5), 55,9% (530/948) tenían un IMC normal (≥18,5-24,9), 18,7% (177/948) tenían sobrepeso (25-29) y 9,7% (92/948) eran obesos (≥ 30). Al margen del estado de la tuberculosis, los pacientes

de bajo peso tuvieron un riesgo de muerte mucho mayor en comparación con los que tenían un IMC normal al inicio del estudio (HRa: 2,9; 95% *CI*: 1,5 – 5,7; *P* = 0,002).

Conclusiones: Independientemente de la coinfección por TB, el IMC bajo se correlacionó con la mortalidad en pacientes infectados con VIH.

Translated from English version into Spanish by Maria Tacost and María Paula Gorgones, through

TRANSLATORS
WITHOUT BORDERS
